# Advances in understanding and managing bullous pemphigoid

**DOI:** 10.12688/f1000research.6896.1

**Published:** 2015-11-20

**Authors:** Cathy Y. Zhao, Dedee F. Murrell

**Affiliations:** 1 Department of Dermatology, St George Hospital, Kogarah, Sydney, NSW 2217, Australia; 2 University of New South Wales, Kensington, Sydney, NSW 2052, Australia

**Keywords:** Bullous pemphigoid, blisters, urticarial plaques, pruritus

## Abstract

Bullous pemphigoid (BP) is the commonest subtype of autoimmune blistering disease in most countries of the world. It occurs most frequently in elderly patients and is characterised clinically by large, tense blisters in the skin preceded by urticarial plaques and pruritus. Immunopathologically, it is characterised by autoantibodies directed against the 180 kD antigen (BP180) and the 230 kD antigen (BP230). New knowledge regarding BP is being continually uncovered. This article reviews the recent advances in BP, including newer diagnostic tests, standardised outcome measures and emerging therapeutic options, as well as the evidence supporting their use.

## Introduction

Bullous pemphigoid (BP) is the commonest subtype of autoimmune blistering disease (AIBD), a rare but potentially fatal group of skin diseases. BP usually affects the elderly and has an incidence of 12.1 to 66 new cases per million per year in epidemiological studies conducted in Europe ^1– 4^. Clinically, it can present heterogeneously but typically manifests with large, tense blisters in the skin preceded by urticarial plaques and intense pruritus. Immunopathologically, it is characterised by subepidermal autoantibodies directed against the 180 kD antigen (BP180) and the 230 kD antigen (BP230), two components of adhesion complexes promoting dermo-epidermal cohesion ^5^.

Medical knowledge regarding BP has progressed considerably in recent years. An important area of progress is newer BP laboratory testing methods, allowing a faster, cheaper and more feasible diagnosis of BP to be established. Another important area is the validation of BP outcome measures, allowing more accurate assessment of disease severity, facilitating the optimal therapy choice and dosage to be administered, and true disease responsiveness to be monitored. As BP usually affects the elderly (over 70 years of age), therapy choice is complicated and needs to be tailored to suit this frail population, balancing between efficacy, practicality and safety. Insights into BP therapies, especially systemic antibody-modulating agents, have increased significantly with regard to both efficacy and safety. This article reviews the recent advances in BP, including diagnostic techniques, outcome measures and therapeutic options, as well as the evidence supporting their use.

## Diagnosis

The diagnosis of BP is based on a combination of clinical, histopathological and immunological criteria ^6^. In the setting of tense bullae with dermal-epidermal separation on histology, or of prodromal separations and positive direct immunofluorescence (DIF) for IgG or C3, the diagnosis of BP can be made if three of the following four criteria are present: age of more than 70 years, absence of atrophic scars, absence of mucosal involvement, and absence of predominant bullous lesions on the neck and head ^7, 8^. This has a sensitivity of 90%, specificity of 83%, and positive predicative value of 95% when validated using immunoelectron microscopy and a sensitivity of 86%, specificity of 90%, and positive predicative value of over 95% when validated using immunoblotting as the gold standard. Therefore, it is recommended to perform a DIF and serological analysis to exclude a BP in all pruritic skin lesion patients who are at least 65 years old ^9^.

The diagnosis of BP may be further confirmed by the characterisation of circulating autoantibodies by using methods such as indirect immunofluorescence (IIF), enzyme-linked immunosorbent assay (ELISA), Western blotting and immunoprecipitation. Developed in the 1980s, IIF is the most commonly used method of autoantibody characterisation, which has a sensitivity of 60–80% in detecting IgG autoantibodies that typically bind to the epidermal side of the salt-split human skin ^10^. The substrate may be obtained commercially or alternatively prepared in the laboratory, the latter of which has the disadvantage of being potentially very time-consuming ^11^. On the other hand, introduced in 2002, ELISA for BP using recombinant protein of BP180 NC16 (the extracellular domain harbouring immunodominant epitopes of BP autoantibodies) is a more sophisticated method of autoantibody characterisation ^12^. It has the advantages of allowing multiple-sample testing, is easily reproducible, and provides a quantitative analysis ^13^. Various validation studies have shown the sensitivity of the commercially available BP180 ELISAs to be 70–90%, which increases when using the NC16A domain and other extracellular portions of BP180 or BP230 together ^6, 12, 14, 15^. However, ELISA has two disadvantages: the cost is high and the recombinant proteins used may not contain all of the epitopes present *in vivo*. In addition, autoantibodies to BP180 and BP230 may be found usually in low levels by ELISA in about 7% of patients with other unrelated diseases or in healthy subjects ^16^. Western blotting and immunoprecipitation are less commonly used given that these methods are often tedious and time-consuming, and have low availability.

Developed in Germany in 2012, the BIOCHIP IF mosaic is a new IIF method for the diagnosis of AIBDs and allows both antibody screening and antigen-specific substrate testing in a single miniature incubation field. So far, four validation studies have been performed, showing that for BP180 testing, it has good sensitivities, varying from 77–100%, and even superior specificities of 84–100% ( Table 1) ^17– 20^. However, for BP230, the sensitivities were much lower, varying from 39–94%, although the specificities were generally good (100% in three studies) ^18– 20^. These cumulative findings suggest that the BIOCHIP IF mosaic in detecting BP180 autoantibodies may be comparable to ELISA, and therefore may be used as a faster and cheaper screening test for patients with suspected BP. However, given the low sample size of the studies, the validities of the findings are compromised. A future study with large sample size and inter-rater reliability evaluation may be indicated for the BIOCHIP mosaic IF test.

**Table 1.** Validation studies evaluating the specificity and sensitivity of biochip indirect immunofluorescence for bullous pemphigoid.

Reference	Country	Number of patients with BP	Gold standard	BP180		BP230	
				Sensitivity	Specificity	Sensitivity	Specificity
Tampoia *et al.* ^20^	Italy	36	ELISA	85%	100%	44%	100%
van Beek *et al.* ^18^	Germany	42	ELISA	100%	98%	55%	100%
Zarian *et al.* ^19^	Italy	18	ELISA	83%	100%	39%	100%
Chiang *et al.* * ^17^	Australia	18	ELISA	77%	84%	94%	63%

*Study in progress. ELISA, enzyme-linked immunosorbent assay.

## Outcome measures

The standardisation of BP outcome measures is important for the progress of BP treatment development, as it allows the direct comparison and meta-analysis of results from different clinical trials. Formed in 2008, the BP Definitions Group consists of many worldwide AIBD experts ^21^. Over a period of 2 years, the group held seven consensus meetings to establish definitions for the various stages of BP disease activity, therapeutic end-points and the first BP-specific severity outcome measure, called the Bullous Pemphigoid Disease Area Index (BPDAI) ^22^. The BPDAI consists of two components: objective and pruritus. Its objective component has up to 360 points and includes blisters or erosions, urticarial or erythematous lesions, and mucosal involvement, each worth up to 120 points. Its separate subjective pruritus component has up to 30 points and takes into account the subjective severity of pruritus in the last 24 hours, week and month. The BPDAI has been validated in terms of its sensitivity to change, accuracy, and external validity by two separate studies ^23, 24^. The studies showed that the BPDAI correlated well with the patient’s erythematous/eczematous/urticarial skin surface, number of daily new blisters, and anti-BP180 titres tested by using ELISA, but not with the anti-BP230 titres. The BPDAI’s inter-rater and intra-rater reliabilities have also been reported to be excellent, and high intra-class correlation coefficients were detailed by a conference abstract summarising their preliminary results ^25^. Future studies to evaluate the interpretability and cross-cultural validity of BPDAI may be indicated in order to complete its validation according to the COSMIN (Consensus-Based Standards for the Selection of Health Status Measurement Instruments) checklist manual ^26^.

Other than disease severity, the BP patient’s quality of life (QOL) may be measured by the Autoimmune Bullous Disease Quality of Life (ABQOL) and Treatment of Autoimmune Bullous Disease Quality of Life (TABQOL) questionnaires. These two questionnaires were developed in 2013, as dermatology disease-specific QOL measures were shown to be more sensitive to changes in disease status than generic QOL measures ^27^. Furthermore, these two questionnaires have been validated as having acceptable levels of construct validity, internal consistency and test-retest reliability, leading to their expansion and further validations in other languages ^21^.

## Treatment

The treatment of BP should be aimed at decreasing blistering formation and pruritus, promote the healing of blisters and improve QOL while having a minimally adverse profile ^28^. The therapeutic options for BP have been transformed significantly in the past decade. Topical clobetasol propionate 0.05% (40 grams per day) has been shown to be superior to oral prednisolone (0.5 mg/kg per day) in terms of overall survival, disease control and adverse event profile for patients with extensive BP. It is equally effective for patients with moderate BP, as shown in a randomised controlled trial (RCT) ^29^. For this reason, topical clobetasol has taken over the previous benchmark of BP therapy, oral corticosteroids, as the first-line treatment for BP. This is supported by the consensus statement from the European Dermatology Forum in collaboration with the European Academy of Dermatology and Venereology ^28, 30^. However, topical clobetasol has the disadvantages of poor practicality in bedridden patients, higher rates of incompliance, and poor accessibility in certain countries. In these cases, oral prednisolone (0.5–1 mg/kg per day), despite its significantly worse adverse event profile, is recommended as the initial therapy instead ^29, 31^.

The use of immunosuppressive medications for BP has been controversial. Their efficacy is generally inconclusive with significant potential adverse events, given prolonged use ^31^.

The most well-established immunosuppressive medication is azathioprine, a purine analogue, typically given at 0.5–2.5 mg/kg per day, followed by mycophenolate mofetil, a DNA-synthesis inhibitor, and methotrexate, a folate antagonist ^28^. There have been a few RCTs evaluating immunosuppressive agents ^32, 33^. One study found azathioprine to have worse hepatotoxicity than mycophenolate ^32^. However, the evidence for efficacy, measured by disease control and remission, was inconclusive. Furthermore, none of the studies used a placebo, which is a control that may be difficult to justify ethically ^31^. Overall, introducing an immunosuppressive medication depends on several factors, including the efficacy of the first-line topical clobetasol or oral corticosteroids, the patient’s disease extent and co-morbidities, the dermatologist’s experiences, and cost considerations.

In this era of biological therapies, new antibody modulators, including rituximab and omalizumab, have been suggested for the treatment of BP. It was proposed that they would have a more benign adverse event profile and more selective mechanisms of action. Rituximab is a humanised chimeric monoclonal antibody that targets CD20 ^+^ B cells. So far, rituximab has shown promising evidence for other AIBDs such as pemphigus; however, in BP, the evidence for rituximab has been limited to case reports and case series ^34– 36^. In a retrospective case series involving five patients with refractory BP, rituximab was administered as 375 mg/m ^2^ given weekly over 4 weeks, resulting in complete remission in three of the five patients, and partial remission in one patient ^34^. However, one other patient died shortly after rituximab therapy. Despite the potential serious adverse events of rituximab, its efficacy for refractory BP was implicated. In a more recent retrospective study, rituximab’s role as first therapy was evaluated ^37^. In this study, a group of 13 patients received 4 weekly infusions of rituximab 500 mg along with oral prednisolone, and a group of 19 patients received oral prednisolone only. The study had a follow-up period of 1 year. It found that the rituximab group had significantly higher rates of complete remission (92% versus 53%) and lower rates of mortality (15% versus 37%). However, there were no significant differences in the patient’s mean BPDAI scores or the cumulative oral corticosteroid dose. The study supported the safety and effectiveness of rituximab but was limited by its retrospective nature and its small sample size. A future RCT with a longer follow-up period, larger sample, and a comparison of various rituximab protocols, may be indicated.

Omalizumab is a humanised monoclonal antibody that inhibits the binding of IgE to its receptors. It has been previously used for asthma and chronic urticaria, and is postulated to be effective for BP as IgE antibodies specific for the BP180 autoantigen have been detected in sera and biopsy samples from the majority of patients with BP ^38^. In an uncontrolled case series of six patients with refractory BP, omalizumab was administered subcutaneously in doses of 300–375 mg from fortnightly to every 6 weeks ^39^. Overall, five of the six patients demonstrated clinical improvements from the omalizumab, and the sixth patient terminated treatment because of inter-current comorbidities. Three of the six patients had their BP180 and BP230 autoantibodies measured by ELISA and showed significant reductions after the use of omalizumab. None of the patients had any significant adverse events. Although the study suggested the efficacy of omalizumab, it was limited by its significantly small sample size, its variation in omalizumab dosage between patients, and its use of non-standardised outcome measures. Future RCTs evaluating omalizumab as a potential treatment for BP may be indicated.

## Conclusions

Knowledge regarding AIBD is rapidly advancing. The diagnosis of BP requires a combination of clinical, histopathological and immunological testing via the detection of tissue-bound and circulating autoantibodies, the latter being the main area of development in recent years. Standardised outcome measures such as the BPDAI have been developed to facilitate the comparison and meta-analysis of clinical trial results. This would likely lead to higher-quality clinical studies evaluating BP treatments, especially immunosuppressives and antibody modulators, with the aim of reducing adverse events associated with oral corticosteroids.
